# Modulation of flagellum attachment zone protein FLAM3 and regulation of the cell shape in *Trypanosoma brucei* life cycle transitions

**DOI:** 10.1242/jcs.171645

**Published:** 2015-08-15

**Authors:** Jack D. Sunter, Corinna Benz, Jane Andre, Sarah Whipple, Paul G. McKean, Keith Gull, Michael L. Ginger, Julius Lukeš

**Affiliations:** 1 Sir William Dunn School of Pathology, University of Oxford, Oxford OX1 3RE, UK; 2 Faculty of Sciences, University of South Bohemia, České Budějovice (Budweis) 37005, Czech Republic; 3 Faculty of Health and Medicine, Division of Biomedical and Life Sciences, Lancaster University, Lancaster LA1 4YQ, UK; 4 Institute of Parasitology, Biology Centre, Czech Academy of Sciences, České Budějovice (Budweis) 37005, Czech Republic; 5 Canadian Institute for Advanced Research, Toronto, Ontario, CanadaM5G 1Z8

**Keywords:** Trypanosomes, Morphogenesis, Flagellum attachment zone

## Abstract

The cell shape of *Trypanosoma brucei* is influenced by flagellum-to-cell-body attachment through a specialised structure – the flagellum attachment zone (FAZ). *T. brucei* exhibits numerous morphological forms during its life cycle and, at each stage, the FAZ length varies. We have analysed FLAM3, a large protein that localises to the FAZ region within the old and new flagellum. Ablation of FLAM3 expression causes a reduction in FAZ length; however, this has remarkably different consequences in the tsetse procyclic form versus the mammalian bloodstream form. In procyclic form cells FLAM3 RNAi results in the transition to an epimastigote-like shape, whereas in bloodstream form cells a severe cytokinesis defect associated with flagellum detachment is observed. Moreover, we demonstrate that the amount of FLAM3 and its localisation is dependent on ClpGM6 expression and vice versa. This evidence demonstrates that FAZ is a key regulator of trypanosome shape, with experimental perturbations being life cycle form dependent. An evolutionary cell biology explanation suggests that these differences are a reflection of the division process, the cytoskeleton and intrinsic structural plasticity of particular life cycle forms.

## Abstract (precis)

**Summary:** *Trypanosoma brucei* FLAM3 is a flagellar FAZ protein. Its depletion leads to a reduction in FAZ length, which has different consequences depending on the life cycle stage of the parasite.

## INTRODUCTION

*Trypanosoma brucei* is a unicellular eukaryotic parasite that causes human African trypanosomiasis. *T. brucei* has a complex life cycle, with stages in both a mammalian host and insect vector, and adopts numerous different morphologies, each adapted to the ecological niche the cell is occupying at that given point in the life cycle (Matthews, 2011; Ooi and Bastin, 2013; Sharma et al., 2009).

The distinctive shape of a trypanosome is the result of a crosslinked sub-pellicular corset of microtubules underlying the plasma membrane. Each cell has a single flagellum, which emerges from the flagellar pocket (FP), an invagination of the cell surface at the base of the flagellum. Tethered to the flagellar basal body is the kinetoplast, a mitochondrial DNA complex (Gluenz et al., 2011; Ogbadoyi et al., 2003; Robinson and Gull, 1991; Robinson et al., 1995; Sherwin and Gull, 1989; Verner et al., 2015). There are several categories of kinetoplastid cell form, which are defined by the relative positions of the nucleus and kinetoplast, and by the point at which the flagellum emerges from the cell body (Hoare and Wallace, 1966). *T. brucei* is found either as a trypomastigote with the kinetoplast posterior to the nucleus or as an epimastigote with the kinetoplast anterior to the nucleus. In both cell forms the flagellum is attached to the cell body.

The attachment of the flagellum to the cell body is mediated by a specialised structure termed the flagellum attachment zone (FAZ), a key regulator of cell shape (Robinson et al., 1995; Vaughan et al., 2008; Zhou et al., 2011). During each cell cycle a trypanosome builds a new flagellum and associated FAZ structure, with the distal end of the new FAZ marking the site of cytokinesis furrow ingression (Robinson et al., 1995). The FAZ is a large cytoskeletal structure that connects a cytoplasmic filament to the axoneme in the flagellum through two membranes and consists of three main regions: filaments linking the axoneme and paraflagellar rod (PFR) to the flagellar membrane, attachments between the flagellar and cell body membranes, and a cytoplasmic FAZ filament and associated cortical microtubule quartet (Hayes et al., 2014; Vaughan et al., 2008).

Protein components from all the main regions of the FAZ structure have been identified and characterised. The first FAZ protein identified was FLA1, a transmembrane protein localised to the cell body membrane associated with the FAZ (Nozaki et al., 1996). Subsequently, the transmembrane protein FLA1-binding protein (FLA1BP) was identified, which interacts with FLA1 and localises to the flagellar membrane associated with the FAZ (Sun et al., 2013). Loss of either FLA1 or FLA1BP leads to flagellum detachment and reduction in the lengths of FAZ and the cell body (LaCount et al., 2002; Sun et al., 2013).

A number of monoclonal antibodies specific to the FAZ filament have been produced: elucidation of the antigen for the antibody L3B2 led to the identification of FAZ1 as a FAZ filament protein (Kohl et al., 1999; Vaughan et al., 2008). CC2D has also been identified as a FAZ filament protein (Zhou et al., 2011). Ablation of CC2D causes a detachment of the flagellum along its entire length as well as severe morphological defects, whereas loss of FAZ1 results in flagellum attachment defects characterised by free loops of flagellum and mis-segregation of the nuclei during cell division (Vaughan et al., 2008; Zhou et al., 2011). Recently, a variety of techniques have been used to identify new FAZ proteins (Morriswood et al., 2013; Sunter et al., 2015; Zhou et al., 2015).

We have recently characterised another FAZ protein, ClpGM6 (Tb927.11.1090), which is large with a central core containing many repeats with calpain-like domains in the N- and C-terminal regions (Hayes et al., 2014). ClpGM6 localises to the flagellar side of the FAZ and knockdown of the protein using RNA interference (RNAi) results in dramatic morphological change, whereby cells adopt an epimastigote-like morphology with the kinetoplast anterior or juxtaposed to the nucleus. There is also a shortening of both the cell body and the FAZ, with an increase in the length of the unattached flagellum. Unusually for a FAZ protein RNAi phenotype, cells depleted of ClpGM6 were viable and proliferated in the epimastigote-like form for many generations (Hayes et al., 2014). Despite the increasing number of identified FAZ proteins, we have little information about the interactions between these proteins, the order of their assembly into the FAZ and whether they form discrete sub-complexes with specific functions.

Our three laboratories independently identified a flagellum-associated protein. Concurrent with our studies, this protein (FLAM3; Tb927.8.4780) was identified in a procyclic form (PCF) flagellum proteome and shown to localise to the FAZ within the flagellum, with its knockdown leading to flagellum detachment (Rotureau et al., 2014). Here, we present our analysis of FLAM3 localisation and RNAi phenotype in both PCF and bloodstream form (BSF) cells, which confirms some aspects of the previous work and extends it in others. We show, as described previously, that FLAM3 localises to the flagellum; however, FLAM3-depleted PCF cells undergo a transformation in shape from a trypomastigote to an epimastigote-like form. Loss of FLAM3 recapitulates the morphological phenotype we observed with ClpGM6 ablation. Moreover, we show that there is interdependency between FLAM3 and ClpGM6 protein expression level: the specific depletion of FLAM3 protein causes a concomitant loss of ClpGM6 and the depletion of ClpGM6 leads to a loss and redistribution of FLAM3.

## RESULTS

### Identification and localisation of FLAM3

**Fig. 1.**
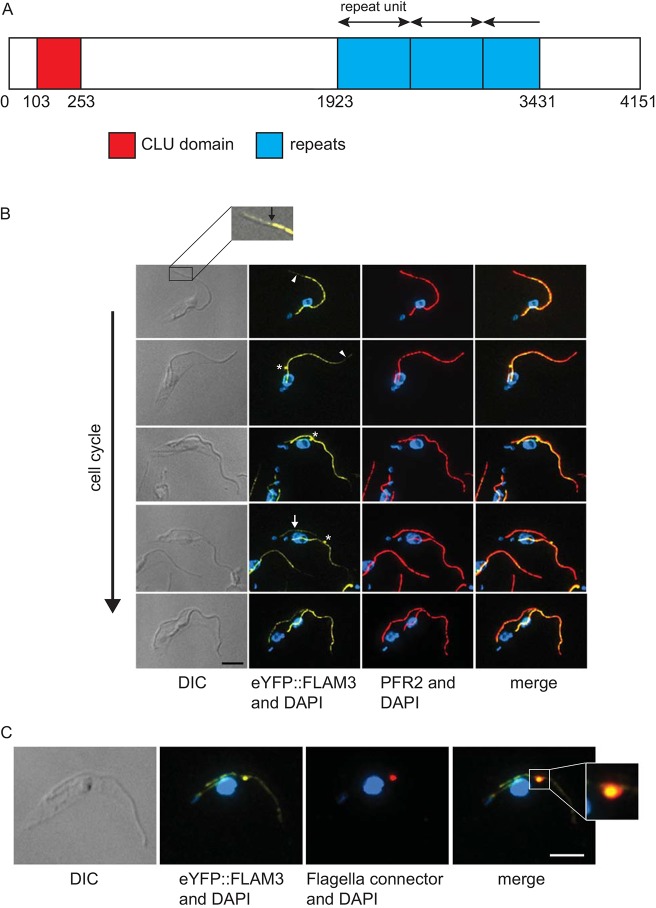
**Schematic of FLAM3 protein and FLAM3 localisation.** (A) Schematic of FLAM3 protein. The predicted CLU domain is in red and the domain containing the repeats (∼2.7×567 aa) in blue. (B) Cytoskeletons at different stages of the cell cycle expressing eYFP::FLAM3 (green), stained for PFR (red) and DNA (blue). White arrowheads indicate the region of the unattached flagellum where the FLAM3 signal is weaker. Asterisks indicate the FLAM3 signal at the flagella connector. The white arrow shows the weaker FLAM3 signal in the new flagellum. The inset shows the anterior end of the cell and the section of unattached flagellum with a weaker eYFP::FLAM3 signal in the unattached free flagellum (indicated by an arrow). Scale bar: 5 µm. (C) Colocalisation of eYFP::FLAM3 (green) with the flagella connector antigen detected by the monoclonal antibody AB1 (red). Scale bar: 5 μm.

FLAM3 (Tb927.8.4780) has previously been identified in both PCF and BSF flagellar proteomes, and its function was examined in PCFs (Price et al., 2012; Rotureau et al., 2014). FLAM3 was of separate interest to our three labs for several reasons, including the presence of ∼1.7 kb (567 amino acids) repeats towards its C-terminus, which show similarity with the 68 amino acid repeats in ClpGM6 and a CLU (clustered mitochondria) domain at the N-terminus (Fig. 1A). Here, we present our combined and complementary study regarding the function of FLAM3 in both PCFs and BSFs.

To determine the localisation of FLAM3, *T. brucei* SMOXP9 cells with an allele of FLAM3 tagged at the N-terminus with eYFP (eYFP::FLAM3) were created. eYFP::FLAM3 localised to the flagellum and the signal partially overlapped with the paraflagellar rod, an extra-axonemal structure; however, eYFP::FLAM3 was not evenly distributed along the length of the flagellum (Fig. 1B). The FLAM3 signal was stronger in the part of the flagellum that was attached to the cell body by the FAZ, and much reduced in the unattached flagellum that extends beyond the anterior end of the cell body (Fig. 1B). Moreover, FLAM3 localisation depended on the stage of the cell cycle, with a distinct difference in eYFP::FLAM3 intensity between the old and new flagellum in cells with two flagella; the signal was stronger in the old flagellum than in the growing new flagellum, suggesting that FLAM3 continues to be integrated into the FAZ beyond the initial assembly of the FAZ structure. FLAM3 also localised to a structure at the tip of the growing flagellum that is likely to be the flagella connector (Fig. 1B). The monoclonal antibodies AB1 and MPM2 detect flagella connector components (Briggs et al., 2004; Davidge et al., 2006) and colocalised with the eYFP::FLAM3 signal (Fig. 1C, data not shown).

### FLAM3 RNAi dramatically changes cell shape

**Fig. 2.**
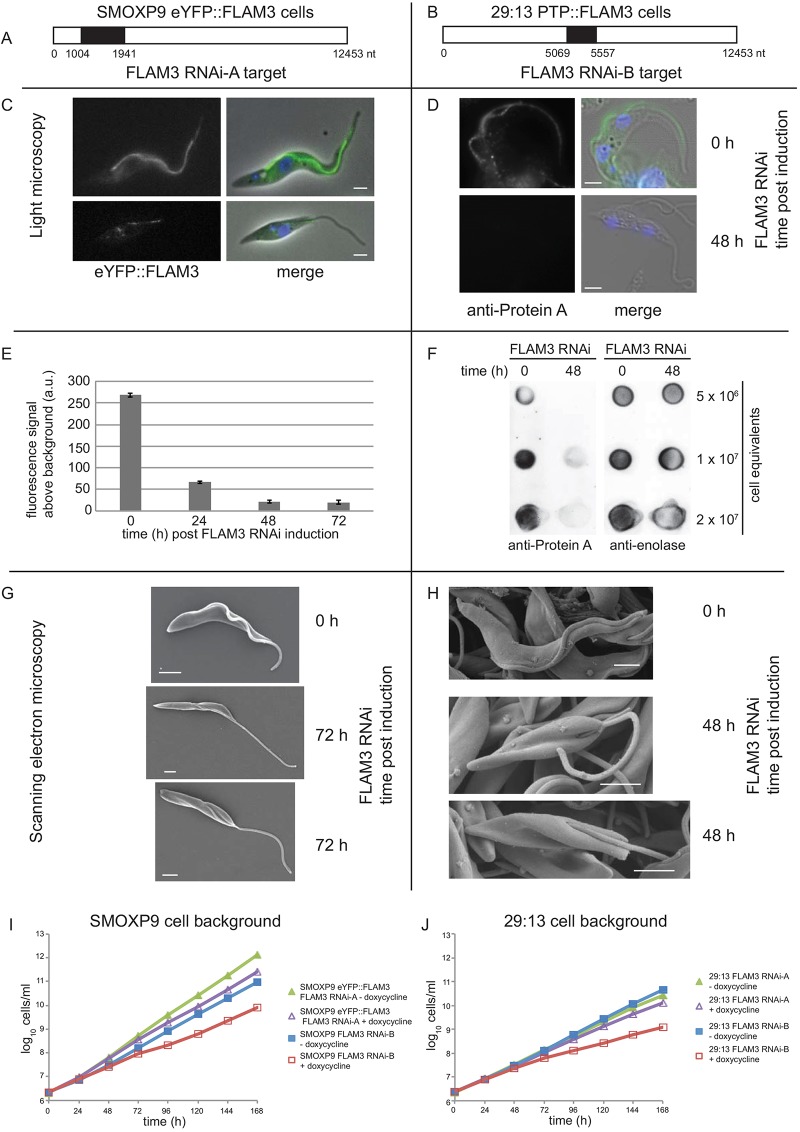
**FLAM3 RNAi causes a dramatic change in cell morphology.** (A,B) Schematics of *FLAM3* gene showing the region amplified for the FLAM3 RNAi-A and RNAi-B plasmids in black. (C) Cells expressing eYFP::FLAM3 (green) with the DAPI stained DNA (blue) before and after 48 h of FLAM3 RNAi induction with the FLAM3 RNAi-A plasmid. (D) Immunofluorescence of the 29:13 cells expressing PTP::FLAM3 before and after 48 h of FLAM3 RNAi induction with the FLAM3 RNAi-B plasmid. PTP::FLAM3 (green) was detected with antibody against protein A; DNA was stained with DAPI (blue). (E) Knockdown of the eYFP::FLAM3 signal measured by flow cytometry during FLAM3 RNAi induction. The median fluorescent signal from three independent inductions was quantified and plotted (±s.d.). (F) Dot blots, using antibody against protein A, of 29:13 FLAM3 RNAi-B cells during RNAi induction. A series of cell equivalents were dotted onto a membrane as indicated and anti-enolase antibody was used as a loading control. (G,H) SEM images of SMOXP9 FLAM3 RNAi-A and 29:13 FLAM3 RNAi-B cells before and after 48 h of FLAM3 RNAi induction. The length of the attached flagellum is reduced in the induced cells. (I) Cumulative growth of SMOXP9 cells with either FLAM3 RNAi-A (green/purple) or FLAM3 RNAi-B (red/blue) plasmid with (purple/red) or without (green/blue) doxycycline induction. The SMOXP9 FLAM3 RNAi-A cell line contains an endogenously eYFP-tagged allele of FLAM3. (J) Cumulative growth of 29:13 cells with either FLAM3 RNAi-A (green/purple) or FLAM3 RNAi-B plasmid (red/blue) with (purple/red) or without (green/blue) doxycycline induction. All scale bars: 2 µm.

FLAM3 function was examined by using inducible RNAi knockdown, with the FLAM3 RNAi phenotype analysis carried out simultaneously in different labs; hence, a variety of cell lines and RNAi plasmids were used. Combining these complementary studies has enabled a thorough investigation of the FLAM3 phenotype. Two different parental cell lines (SMOXP9 and 29:13) were used for the phenotype analysis, both carrying tetracycline-inducible FLAM3 RNAi constructs that were based on the p2T7-177 plasmid, but targeting different regions of the FLAM3 gene (FLAM3 RNAi-A and FLAM3 RNAi-B – Fig. 2A,B). In addition, both cell lines contained an endogenously tagged allele of FLAM3 to validate protein depletion (the N-terminal eYFP tag for the SMOXP9 strain and the N-terminal protein-C–TEV–protein-A (PTP) tag for the 29:13 stain). Initially, RNAi-A was used in K.G.’s and M.L.G.’s labs, and RNAi-B in J.L.’s laboratory.

On RNAi induction the knockdown of FLAM3 protein was examined by imaging the loss of the native eYFP::FLAM3 or PTP::FLAM3 signals by immunofluorescence using an antibody against protein A in the respective cell lines (Fig. 2C,D). The loss of eYFP::FLAM3 was measured using flow cytometry during FLAM3 RNAi induction with the fluorescent signal dropping by ∼90% (Fig. 2E). The reduction, visualised by microscopy, was also confirmed by western blotting by using an anti-GFP antibody and dot blots using antibody against protein A (supplementary material Fig. S1A, Fig. 2F).

On FLAM3 RNAi induction there was a dramatic change in cell morphology: before induction, all cells with a single flagellum were of a trypomastigote form but after induction many cells displayed an epimastigote-like form with a long unattached flagellum and the kinetoplast juxtaposed or anterior to the nucleus (Fig. 2C, supplementary material Fig. S2A). After 72 h of induction, over 90% of 1 kinetoplast 1 nucleus (1K1N) 29:13 FLAM3 RNAi-B cells had an epimastigote-like morphology (supplementary material Fig. S2B). Importantly, this effect was observed with both the SMOXP9 and 29:13 RNAi cell lines and both FLAM3 RNAi plasmids (supplementary material Fig. S2A). The epimastigote-like appearance was confirmed by scanning electron microscopy (SEM) of the FLAM3 RNAi-induced cells (Fig. 2G,H).

The growth rates of both the SMOXP9 FLAM3 RNAi-A and 29:13 FLAM3 RNAi-B cell lines were measured during FLAM3 RNAi induction (Fig. 2I,J). Despite both FLAM3 RNAi cell lines showing a reduction in FLAM3 expression and change in cell shape (Fig. 2, supplementary material Fig. S2A), a difference in growth rate was observed. In the SMOXP9 eYFP::FLAM3 FLAM3 RNAi-A cell line there was only a slight reduction in growth over the induction (Fig. 2I). In the 29:13 FLAM3 RNAi-B cell line the growth rate began to drop after 48 h of induction and cells grew very slowly from that point onwards (Fig. 2J).

The above FLAM3 RNAi growth rates were estimated in cells that had been derived from two different parental strains containing different RNAi plasmids. To investigate the effect of the different RNAi plasmids on cell growth, both were integrated into the 29:13 cell line; the growth rate after doxycycline induction was measured with the doubling time calculated using non-linear regression (Fig. 2J). Induction of the FLAM3 RNAi-B plasmid had a stronger effect on cell growth than the FLAM3 RNAi-A plasmid: the former increased the doubling time from 12 h (r^2^=0.99) to 22.7 h (r^2^=1.0), whereas the latter increased the doubling time from 13.3 h (r^2^=0.99) to 14.8 h (r^2^=0.99). This effect was also observed when these RNAi plasmids were integrated into the SMOXP9 cell line, with the induction of the FLAM3 RNAi-B plasmid increasing the doubling time from 10.4 h (r^2^=0.99) to 12.6 h (r^2^=1.0) and the FLAM3 RNAi-A plasmid increasing the doubling time from 8.6 h (r^2^=1.0) to 9.5 h (r^2^=1.0) (Fig. 2I). The SMOXP9 FLAM3 RNAi cell lines grew faster than the equivalent 29:13 FLAM3 RNAi cell lines (Fig. 2I,J); this is unsurprising as the parental SMOXP9 cells have previously been shown to grow quicker than 29:13 cells (Poon et al., 2012).

**Fig. 3.**
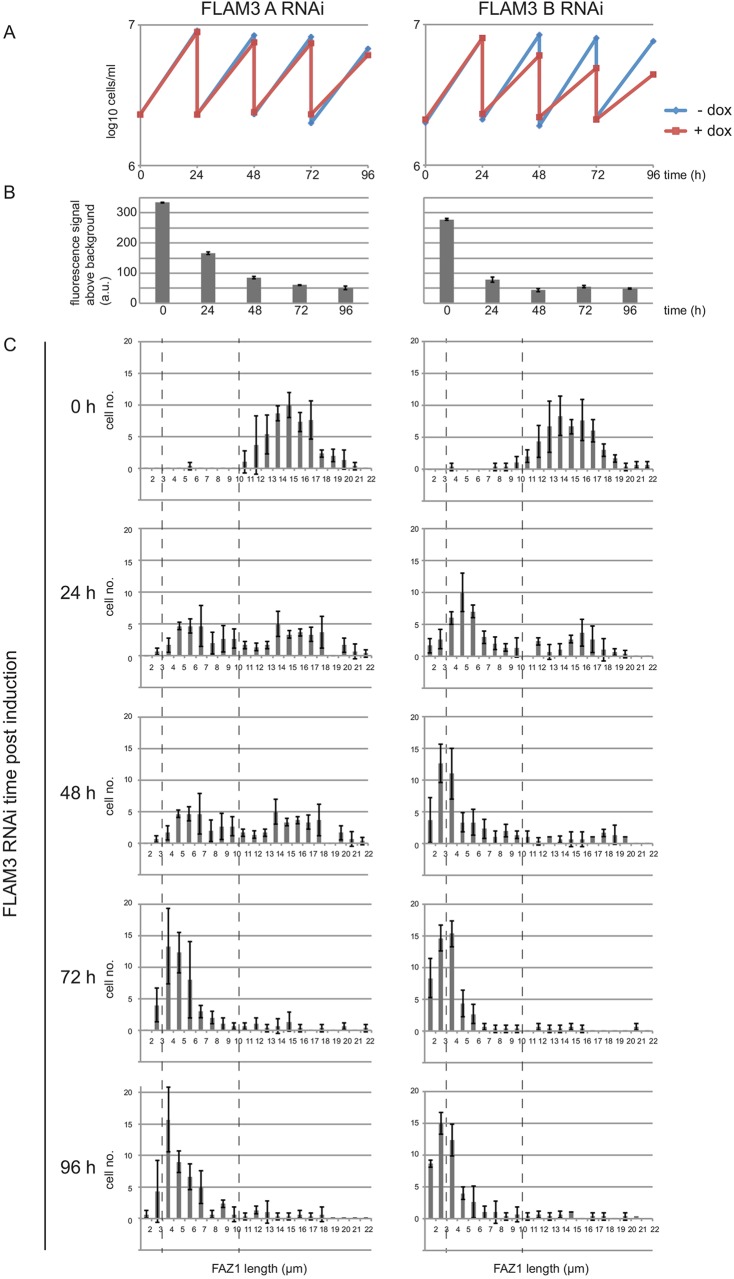
**FAZ length correlates with the growth rate of the epimastigote-like cells.** (A) Growth curves of 29:13 cells with either the FLAM3 RNAi-A or B plasmid with or without doxycycline induction. (B) Knockdown of eYFP::FLAM3 signal in 29:13 FLAM3 RNAi-A and RNAi-B cells measured by flow cytometry during a FLAM3 RNAi induction. The median fluorescent signal from three independent inductions was plotted (±s.d.). (C) Histograms of FAZ1 lengths from 1K1N detergent extracted cytoskeletons during FLAM3 RNAi induction for both RNAi plasmids (the lengths of 50 FAZ1 measured per time point from three independent experiments; error bars indicate ±s.d.).

We hypothesised that the growth defect observed in 29:13 FLAM3 RNAi-B cells (Fig. 3A) was due to the FAZ dropping below a certain threshold of length that is required to support cytokinesis, and that 29:13 FLAM3 RNAi-A cells were able to proliferate as the length of the FAZ remained above this threshold. We examined the relative loss of FLAM3 in the 29:13 FLAM3 RNAi-A and B cells by integrating the eYFP::FLAM3 tagging plasmid into both of them and measuring the loss of eYFP:FLAM3 by flow cytometry after FLAM3 RNAi induction (Fig. 3B). The FLAM3 RNAi-B plasmid resulted in a more-rapid loss of the eYFP::FLAM3 signal and dropped to a slightly lower level than that of the RNAi-A plasmid. We then determined the minimum length of FAZ that is required, by measuring FAZ1 length during a FLAM3 RNAi induction in 1K1N 29:13 FLAM3 RNAi-A and B cytoskeletons (Fig. 3C). The mean FAZ1 length for both cell lines dropped after induction and, as expected, the reduction was greater in the FLAM3 RNAi-B cytoskeletons (Fig. 3C). There were three distinct sub-populations of FAZ1 length observed into which the FLAM3 RNAi cytoskeletons could be divided. The non-induced cytoskeletons had a FAZ1 length of 10–21 µm with the induced populations split into FAZ1 lengths of either 1–3 µm or 3–10 µm (Fig. 3C). The majority of the FLAM3 RNAi-A cytoskeletons never dropped below 3 µm, whereas by 48 h almost one-third of the FLAM3 RNAi-B cytoskeletons had a FAZ1 length of less than 3 µm, and after this time point the FLAM3 RNAi-B cells grew very slowly. These data suggest that the critical minimum FAZ length is ∼3 µm, below which the FAZ is unable to support cytokinesis.

### FLAM3 RNAi mirrors ClpGM6 RNAi

**Fig. 4.**
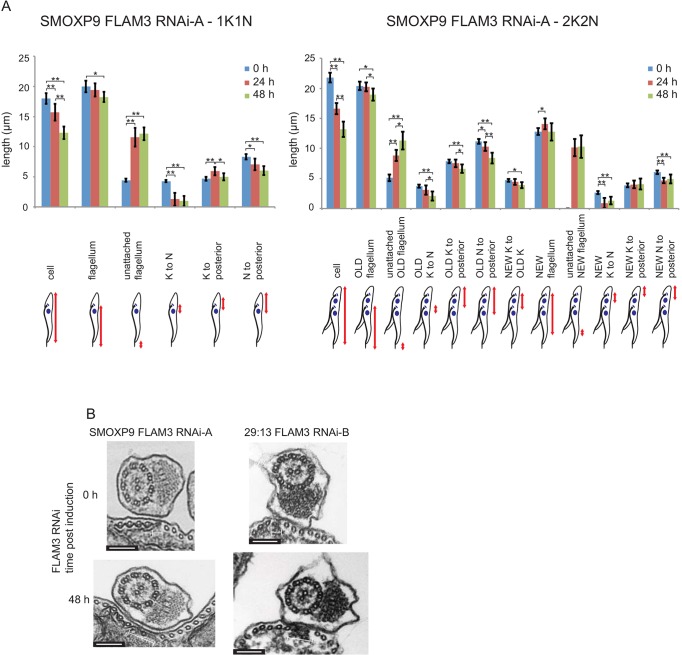
**Similarities of the FLAM3 RNAi phenotype to ClpGM6 RNAi phenotype to the ClpGM6 phenotype.** (A) Graphs showing morphological measurements represented by the cartoons for 1K1N and 2K2N cells during a FLAM3 RNAi timecourse in the SMOXP9 FLAM3 RNAi-A cell line. Measurements were taken every 24 h for 30 1K1N and 30 2K2N cells, and the lengths were plotted; error bars represent ±95% confidence levels. Morphometric measurements were statistically evaluated using SPSS software and either a one-way ANOVA or, when required, the non-parametric Kruskal–Wallis test. *P≤0.05 and **P≤0.005 indicate statistically significant differences between two groups. K, kinetoplast; N, nucleus. (B) TEM images of cross sections across the flagellum and FAZ in SMOXP9 FLAM3 RNAi-A and 29:13 FLAM3 RNAi-B cells before and after 48 h of FLAM3 RNAi induction. The width of the FAZ region (indicated by the white bar) is increased in the induced cells. Scale bars: 150 nm.

The change in cell morphology from a trypomastigote form to an epimastigote-like form was reminiscent of the morphological changes observed on ClpGM6 knockdown (Hayes et al., 2014). An analysis of the changes in cell shape was undertaken in M.L.G.’s laboratory, by measuring a variety of cell parameters on both 1K1N and 2K2N cells during induction of FLAM3 RNAi when using SMOXP9 FLAM3 RNAi-A cells (Fig. 4A).

For 1K1N cells the most striking morphometric change observed during induction was the substantially increased length of the free, unattached flagellum [4.4±0.7 µm (mean±s.d.) (*n*=30) in uninduced cells vs 12.1±2.8 µm (*n*=30) in induced cells], which was not reflected in concomitant changes in flagellum length [20.0±2.6 µm (*n*=30) in uninduced cells vs 18.3±2.2 µm (*n*=30) in induced cells], implying that the cells had a shorter FAZ. A shorter FAZ was consistent with the reduction in cell body length [18.0±2.4 µm (*n*=30) in uninduced cells vs 12.3±2.8 µm (*n*=30) in induced cells]. A further change observed was the reduction in kinetoplast-nucleus distance with the kinetoplast next to or anterior to the nucleus [4.3±0.6 µm (*n*=30) in uninduced cells vs 1.0±2.3 µm (*n*=30) in induced cells]. The changes in 1K1N cells during FLAM3 RNAi were reflected in 2K2N cells (Fig. 4A) and, overall these results match those observed on ClpGM6 knockdown (Hayes et al., 2014).

To confirm the phenotype similarities between ClpGM6 and FLAM3 knockdown cells, the ultrastructure of both the SMOXP9 and 29:13 FLAM3 RNAi cell lines was studied by using thin-section transmission electron microscopy of whole cells. Transverse sections of the cell showing the flagellum and the FAZ were analysed: after 48 h of FLAM3 RNAi induction the gap between the sub-pellicular microtubules and the microtubule quartet – the location of the FAZ filament – was much wider than in uninduced cells, mirroring the result seen with ClpGM6 RNAi (Fig. 4B). The change in morphology on FLAM3 knockdown was not accompanied by the expression of the epimastigote-specific surface marker *T. brucei* alanine-rich protein (BARP) matching the ClpGM6 RNAi phenotype (supplementary material Fig. S1B,C) (Hayes et al., 2014).

### FLAM3 is necessary to maintain ClpGM6 levels

**Fig. 5.**
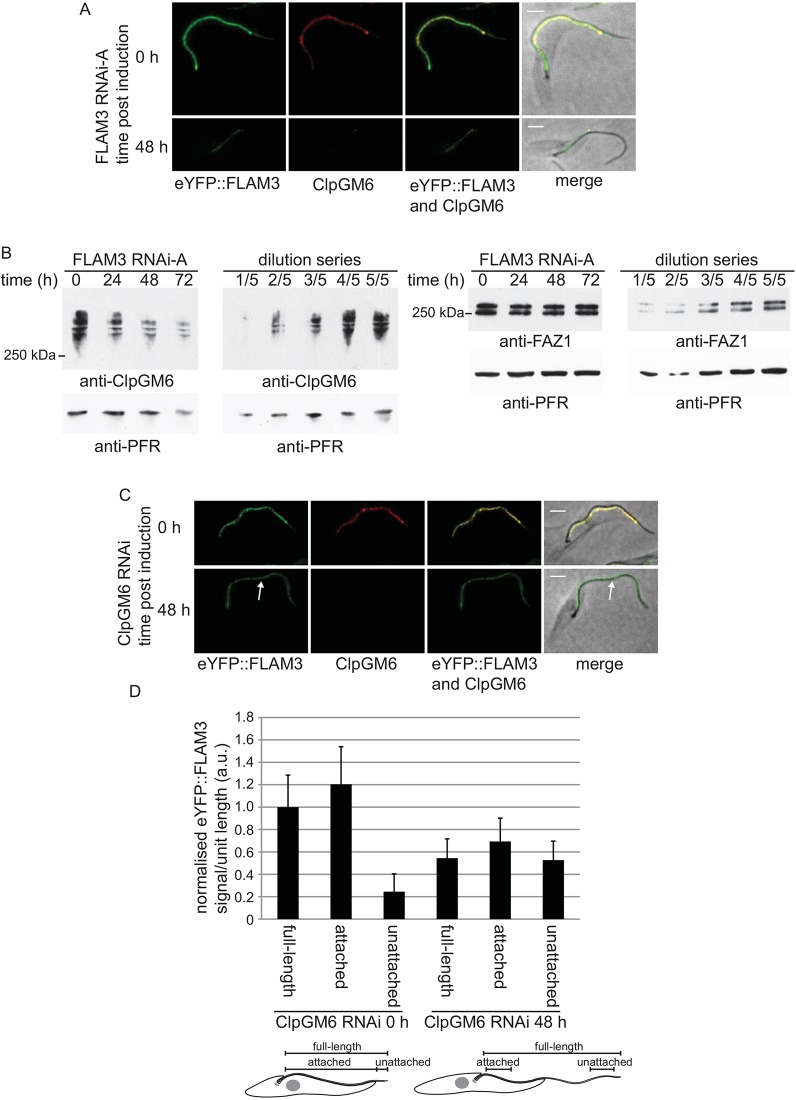
**Interdependency of ClpGM6 and FLAM3 expression.** (A) Cytoskeletons that express eYFP::FLAM3 (green) were stained for ClpGM6 (red) before and after 48 h of FLAM3 RNAi induction. Scale bars: 2 µm. (B) Western blot using the anti-ClpGM6 antibody of whole cells during a FLAM3 RNAi induction. The same samples were run on a separate blot and probed for FAZ1 by using anti-FAZ1 (L3B2) antibody. Anti-PFR (L13D6) antibody was used as a loading control; 2×10^6^ cell equivalents were loaded per lane except in the dilution series where decreasing cell equivalents were loaded as indicated. (C) Cytoskeletons expressing eYFP::FLAM3 (green) were stained for ClpGM6 (red) before and after 48 h of ClpGM6 RNAi induction. White arrow indicates eYFP::FLAM3 present in the unattached flagellum at a similar intensity to the attached flagellum. Scale bars: 2 µm. (D) Quantification of eYFP::FLAM3 signal before and after 48 h of ClpGM6 RNAi induction. Cartoons of a FLAM3 RNAi uninduced cell and an induced cell indicate the regions where the signal intensity was measured. A 20 pixel-wide line was traced along the flagellum, for the full-length of the flagellum, the length of the attached flagellum or the length of the unattached flagellum. The signal intensity of every pixel measured was added up and then divided by the traced length to give the average signal intensity per unit length for that region. These values were then normalised relative to the uninduced full-length value (±s.d.). A representative set of data from one of three independent experiments is shown.

Knockdown of FLAM3 results in a phenotype that is similar to the one described for the loss of ClpGM6; moreover, both proteins are associated with the FAZ region within the flagellum. This led us to investigate the relationship between ClpGM6 and FLAM3 setting up the following experiments, performed in K.G.'s laboratory, using the FLAM3 RNAi-A plasmid. ClpGM6 localisation was analysed by immunofluorescence with the anti-ClpGM6 antibody during FLAM3 RNAi knockdown using the SMOXP9 FLAM3 RNAi-A cells. In cytoskeletons of cells that had adopted an epimastigote-like appearance and in which the eYFP::FLAM3 signal was reduced, there was also a concomitant loss of the ClpGM6 signal (Fig. 5A).

To differentiate between the loss of ClpGM6 protein and its redistribution to elsewhere in the cell, expression of ClpGM6 was investigated by western blotting of whole-cell lysates during FLAM3 knockdown by using the anti-ClpGM6 antibody (Fig. 5B). Induction of FLAM3 RNAi led to a decrease in ClpGM6 protein levels over the first 48 h with no further loss by 72 h, suggesting that the presence of FLAM3 is necessary for the maintenance of ClpGM6 levels and its localisation to the FAZ. However, knockdown of FLAM3 only had a minimal effect on the levels of another FAZ protein, FAZ1, which localises to the cytoplasmic FAZ filament (Fig. 5B, supplementary material Fig. S3A). In cells that had adopted an epimastigote-like morphology during FLAM3 RNAi, the FAZ1 signal appeared more intense than in uninduced trypomastigotes, suggesting that FAZ1 had become concentrated in the shorter FAZ (supplementary material Fig. S3A).

### ClpGM6 ablation causes loss and redistribution of FLAM3

To investigate the effect of ClpGM6 knockdown on FLAM3, the ClpGM6 RNAi plasmid was integrated into the SMOXP9 cell line expressing eYFP::FLAM3. Successful ClpGM6 knockdown was confirmed by loss of the ClpGM6 immunofluorescence signal from cytoskeletons, and by the change to an epimastigote-like cell morphology (Fig. 5C). In the cytoskeletons that had lost ClpGM6 and exhibited an epimastigote-like shape, the localisation of eYFP::FLAM3 was altered; FLAM3 was now evenly distributed along the flagellum with a reduced signal intensity (Fig. 5C). This change in FLAM3 localisation pattern was also observed in ClpGM6-depleted whole cells (supplementary material Fig. S3B). The even distribution of FLAM3 along the flagellum could be the result of (i) redistribution of FLAM3 within the flagellum; (ii) specific loss of FLAM3 from the FAZ region, so the signal now matches that observed in the unattached flagellum; (iii) a combination of (i) and (ii).

To determine which of these explanations was most likely, the signal intensity of eYFP::FLAM3 along the flagellum was analysed. The average intensity per unit length of the eYFP::FLAM3 signal along the flagellum was calculated for different regions of the flagellum in cytoskeletons where ClpGM6 RNAi was not induced or induced for 48 h (Fig. 5D). Cytoskeletons were used for this analysis to focus on the FLAM3 that was stably associated with the assembled FAZ. ClpGM6 knockdown led to an ∼50% decrease in overall FLAM3 signal intensity; however, despite this, FLAM3 signal intensities in ClpGM6-depleted cytoskeletons did not decrease to the levels observed in the unattached part of the flagellum of cytoskeletons in which RNAi of ClpGM6 had not been induced. In this experiment there was only a small reduction in the overall length of the FLAM3 signal following ClpGM6 RNAi [19.6±2.0 µm (*n*=30) vs 18.8±2.6 µm (*n*=30)] (data not shown), these results imply that, during ClpGM6 knockdown, there was both loss of FLAM3 signal intensity and a redistribution of the protein along the flagellum.

To discover whether FLAM3 had been degraded or redistributed to elsewhere in the cell during ClpGM6 RNAi, the eYFP::FLAM3 signal was measured in whole cells where ClpGM6 RNAi had been not induced or induced for 48 h by flow cytometry. Depletion of ClpGM6 led to a ∼30% reduction in eYFP::FLAM3 signal (supplementary material Fig. S3D). Using this same approach, induction of FLAM3 RNAi led to an ∼90% reduction of the FLAM3 signal intensity when measured after 48 h (supplementary material Fig. S3C). This suggests that the presence of ClpGM6 is necessary for the maintenance of FLAM3 levels but not for its localisation to the flagellum.

### FLAM3 is essential in BSF cells

**Fig. 6.**
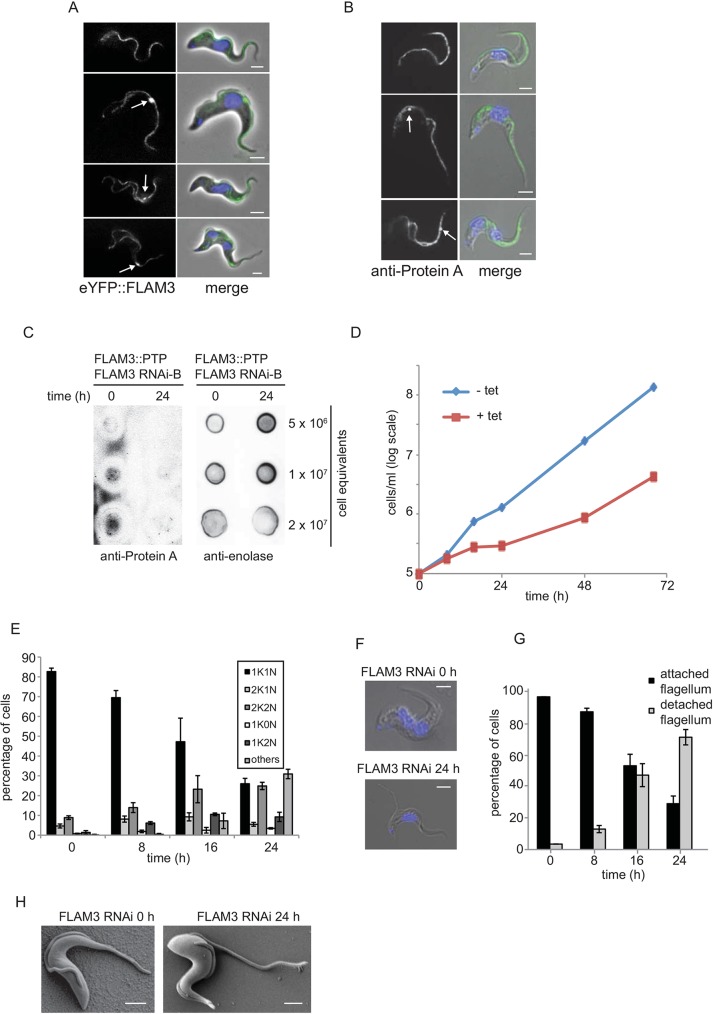
**FLAM3 RNAi in BSF cells causes a reduction in the length of attached flagellum, resulting in a defect in cytokinesis.** (A) BSF cells at different stages of the cell cycle expressing eYFP::FLAM3 (green); DNA was stained with DAPI (blue). White arrows indicate the strong FLAM3 signal at the distal tip of the new flagellum. Scale bars: 2 µm. (B) Immunofluorescence of BSF cells expressing FLAM3::PTP at different stages of the cell cycle. The white arrows indicate the strong FLAM3 signal at the distal tip of the new flagellum. FLAM3::PTP (green) was detected with antibody against protein A; DNA was stained with DAPI (blue). Scale bars: 2 µm. (C) Dot-blot analysis of FLAM3 RNAi cells expressing PTP::FLAM3. A series of equivalent cells was dotted onto a membrane as indicated and the PTP::FLAM3 detected with antibody against protein A. Anti-enolase was used as a loading control. (D) Cumulative growth of SM BSF FLAM3 RNAi cells with (red) or without (blue) addition of tetracycline. Data for one representative clone are shown. The increase in the growth rate of the induced cell line after 48 h is likely to be due to the presence of RNAi refractory cells accumulating in the culture. (E) Analysis of cell cycle stages following FLAM3 depletion. FLAM3 RNAi was induced with tetracycline for 24 h and the mean percentage of each cell category (±s.d.) was plotted. Per time point, ≥200 cells were scored in three independent experiments. (F) SM BSF FLAM3 RNAi cells with the before and after 24 h of FLAM3 RNAi induction; DNA was stained with DAPI (blue). Scale bars: 2 µm. (G) FLAM3 RNAi causes flagellum detachment. FLAM3 RNAi was induced with tetracycline for 24 h, and the mean percentage of cells (±s.d.) with and without flagellum detachment was plotted. Per time point, ≥200 cells were scored in three independent experiments. (H) Representative images of cells before (left panel) and after 24 h of tetracycline induction (right panel). Scale bars: 2 µm. (H) SEM images of SM BSF FLAM3 RNAi cells before and after 24 h of FLAM3 RNAi induction. The attached flagellum is shorter in induced cells. Scale bars: 2 µm.

The localisation of FLAM3 in BSF cells was determined by tagging an endogenous allele of FLAM3 with an N-terminal eYFP tag in the SMOXB4 cell line. eYFP::FLAM3 localised to the FAZ and the signal did not extend beyond the anterior end of the cell into the unattached flagellum (Fig. 6A). In cells with two flagella, a strong punctate signal was observed that was likely to be associated with the distal tip of the growing new flagellum. The localisation of FLAM3 in BSF cells was confirmed by tagging an endogenous copy of FLAM3 at the C-terminus with PTP. Immunofluorescence using antibody against protein A showed that FLAM3 localised to the FAZ with a strong signal associated with the tip of the new flagellum (Fig. 6B).

The BSF experiments were performed in the laboratory of J.L., using the FLAM3 RNAi-B plasmid. To determine whether the epimastigote-like phenotype can be observed in BSF cells, the FLAM3 RNAi-B plasmid was transfected into the BSF single-marker cell line, which had an endogenous allele of FLAM3 tagged with PTP at the N-terminus. When FLAM3 RNAi was induced, loss of FLAM3 protein was confirmed by dot blots of whole-cell lysates using antibody against protein A, showing an almost complete disappearance of FLAM3 after 24 h of RNAi induction (Fig. 6C). The growth rate of the cells after FLAM3 RNAi induction was measured and a growth defect occurred at an earlier time point than in PCFs (Fig. 6D). The cells grew very slowly after RNAi induction when compared with the control cell line.

To further analyse the BSF phenotype, kinetoplasts and nuclei were stained with DAPI and counted during FLAM3 RNAi induction (Fig. 6E). The number of kinetoplasts and nuclei in a cell indicates the cell cycle stage. During 24 h of FLAM3 RNAi induction, the percentage of cells in normal cell cycle stages decreased, with a concomitant increase in multinucleate and other abnormal cell types – including zoids (1K0N) and 1K2N cells; this suggests that the loss of FLAM3 leads to a defect in cytokinesis (Fig. 6E).

In contrast to the PCF FLAM3 RNAi phenotype, no BSF cells were observed in which the kinetoplast was anterior or juxtaposed to the nucleus during FLAM3 knockdown. Furthermore, after loss of FLAM3 the BSF cells had a detached flagellum in contrast to the long unattached flagellum observed on PCF cells (Fig. 6F). Quantification of cells with a detached flagellum during FLAM3 RNAi knockdown indicated a steadily increasing number of these, reaching ∼70% after 24 h of induction (Fig. 6G). To confirm this phenotype, induced FLAM3 RNAi cells were examined by using SEM. After 24 h of induction, cells with a detached flagellum were readily observed (Fig. 6H), which suggests that loss of FLAM3 disrupts FAZ assembly, thereby, causing flagellum detachment.

## DISCUSSION

Here, we have investigated the localisation and function of FLAM3. An earlier study observed localisation of FLAM3 to the FAZ within the flagellum in PCFs and our initial observations confirmed this (Rotureau et al., 2014); however, we noticed some important features that had not been described previously. The signal intensity of the FLAM3 signal was strongest in the FAZ region of the flagellum, with a much-reduced signal extending into the unattached part of the flagellum. This lower level of FLAM3 may represent protein excess that could not be integrated into the FAZ or may mean that FLAM3 has a role in the flagellum beyond the FAZ. In cells with two flagella the signal in the new flagellum was less intense than in the old flagellum. The difference in FLAM3 signal between the new and old flagellum could indicate that additional FLAM3 is integrated into the FAZ once the flagellum is fully assembled to strengthen the connections between the FAZ and the flagellar skeleton. Finally, during the initial stages of flagellum assembly, we observed a strong punctate signal at the tip of the new flagellum, which is likely to be the flagella connector; late in the cell cycle the punctate tip signal was less pronounced. Here, FLAM3 was tagged on its N-terminus with eYFP, whereas in the previous study FLAM3 was tagged on its C-terminus, which may cause the differences in localisation observed (Rotureau et al., 2014). In BSFs FLAM3 also localised to the FAZ; however, there were differences compared to the PCFs. In BSFs the FLAM3 signal did not extend beyond the cell body and there was no distinct difference in signal intensity of FLAM3 in the old and new FAZ.

FLAM3 RNAi PCF cells underwent dramatic morphological change, i.e. from a trypomastigote to an epimastigote-like shape. FLAM3 loss resulted in the shortening of the FAZ with a large increase in the length of the unattached flagellum and the kinetoplast being positioned anterior or juxtaposed to the nucleus. We suggest that the loss of FLAM3 becomes a limiting factor in FAZ assembly, resulting in the construction of a shorter FAZ. The FLAM3 RNAi phenotype was highly reproducible and the morphological change was observed using two different parental strains containing different FLAM3 RNAi plasmids. The growth effect observed during FLAM3 knockdown varied depending on the FLAM3 RNAi plasmid used. The simplest explanation for the difference in growth is the relative penetrance of the FLAM3 knockdown caused by the different plasmids. This mirrors the situation observed with ClpGM6 knockdown, where different ClpGM6 RNAi plasmids produced varying degrees of cell growth defects (Hayes et al., 2014).

The different growth effects of the two FLAM3 RNAi plasmids allowed us to define a minimum length of FAZ required for continued cell division in PCFs. For the majority of the FLAM3 RNAi-A induced cells the FAZ length did not decrease to less than 3 µm, whereas for the FLAM3 RNAi-B induced cells the FAZ length did decrease to less than 3 µm and this coincided with the decrease in growth rate. At a length of less than 3 µm, the FAZ structure is likely to be severely disrupted and, hence, the cells developed cytokinesis defects that resulted in the formation of multi-nucleate monster cells (supplementary material Fig. S2C). This now allows us to begin to define the architectural limits of the PCF cell.

Recently, it has been claimed that loss of FLAM3 led to flagellum detachment in PCF cells (Rotureau et al., 2014). The FLAM3 RNAi phenotype has probably been misinterpreted previously, because our SEM results show no evidence for the flagellum becoming detached during FLAM3 RNAi of PCF cells. Instead, we observed a decrease in the length of the FAZ and a substantial increase in the length of the unattached flagellum, which could give the appearance of flagellar detachment when using light microscopy. The phenotype of FLAM3 ablation matches that observed previously for ClpGM6 RNAi (Hayes et al., 2014). We demonstrated a reciprocal dependency of FLAM3 and ClpGM6 levels. During ClpGM6 RNAi there was a redistribution of the FLAM3 signal within the flagellum in addition to loss of FLAM3 signal intensity. By contrast, the ClpGM6 signal disappeared from the flagellum during FLAM3 RNAi. This pattern suggests a hierarchy of assembly, in which FLAM3 is required for ClpGM6 stability and localisation.

The similar RNAi phenotypes and expression dependencies suggest that FLAM3 and ClpGM6 are functionally related, and mass spectrometry of proteins immunoprecipitated with ClpGM6 revealed the presence of FLAM3 (data not shown). Moreover, orthologues of both FLAM3 and ClpGM6 occur in the protozoan *Bodo saltans*, suggesting that these proteins are found together across the kinetoplastids (Sunter et al., 2015). Strikingly, their domain architectures are also conserved: ClpGM6 with its N-terminal calpain-like domain preceding many copies of a relatively short amino acid repeat (68 amino acids in *T. brucei*) versus the N-terminal CLU domain of FLAM3, which is followed by two to three copies of a much larger amino-acid repeat (567 amino acids in *T. brucei*). With respect to the difference in repeat length, it is perhaps intriguing that the laboratories of K.G. and M.L.G. first identified FLAM3 in homology searches using the ClpGM6 repeat. The importance of the CLU-related domain in flagellum-localised FLAM3 is unclear; the CLU domain is a principal architectural feature recognisable in *Dictyostelium* cluA orthologues. These conserved, soluble proteins are necessary for normal mitochondrial morphology and positioning in diverse eukaryotes (Fields et al., 1998; Logan et al., 2003; Sen et al., 2013), although we would like to point out the recent publication by Gao and colleagues, reporting evidence of a partial cytoskeletal association of CLUH, the mammalian orthologue of cluA (Gao et al., 2014).

In supplementary material Fig. S4 we outline a model whereby FLAM3 forms part of a complex with ClpGM6, which is then integrated into the flagellum. If either ClpGM6 or FLAM3 were lost, the partially formed complex would be degraded. Complex formation might only require a proportion of FLAM3 and further FLAM3 could be integrated into the flagellum at later time points, providing an explanation for the difference in FLAM3 signal between the old and new FAZ. Loss of ClpGM6 would result in degradation of the complex and, therefore, loss of FLAM3. However, because not all FLAM3 would be integrated into the complex, the remainder would then be integrated along the length of the flagellum (supplementary material Fig. S4).

**Fig. 7.**
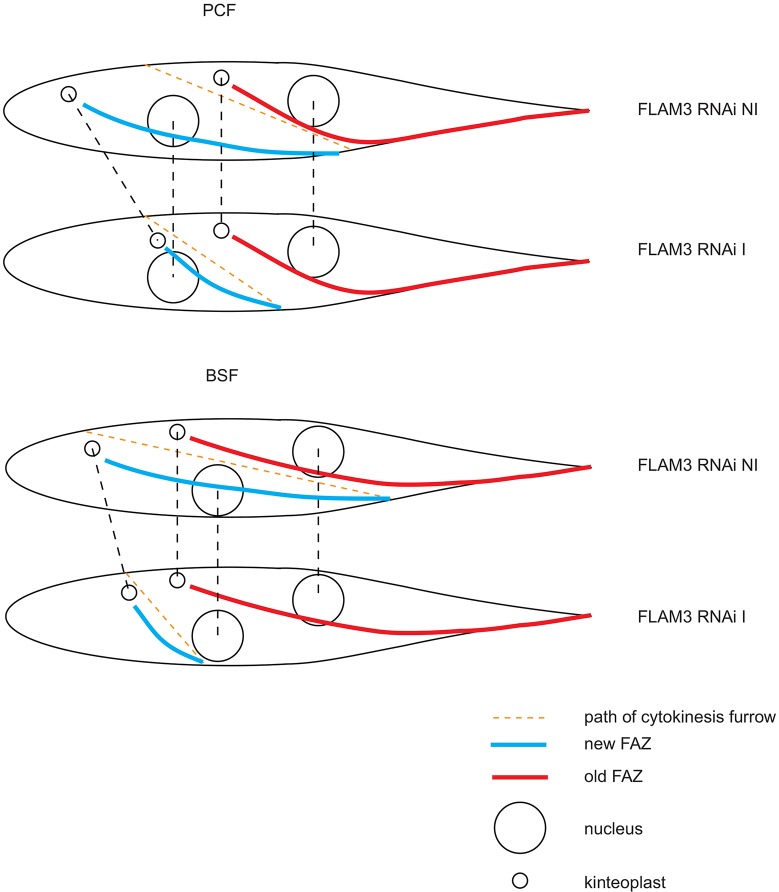
**A model that, following FLAM3 RNAi, shows the effect of a short FAZ on the first cell division post-induction in both PCF and BSF *T. brucei*.** In PCF cells the inter-nuclear distance is unaffected by FLAM3 RNAi, whereas the inter-kinetoplast distance is reduced. Therefore, the posterior kinetoplast is juxtaposed to the posterior nucleus. The shorter FAZ changes the position of the cytokinesis furrow, which results in the production of a daughter cell with a short cell body, a long unattached flagellum and the kinetoplast juxtaposed to the nucleus. In BSF cells the inter-nuclear distance is unaffected by FLAM3 RNAi but the inter-kinetoplast distance is reduced; however, the separation of the nucleus is not as great as in PCF cells, so the posterior kinetoplast is not juxtaposed to the posterior nucleus. The shorter FAZ changes the position of the cytokinesis furrow, which ends closer to the existing posterior in BSF cells and, therefore, results in the production of a zoid.

Knockdown of FLAM3 in both BSFs and PCFs inhibits assembly of the FAZ but this results in striking differences in the morphological phenotype of the cells. In BSFs the inhibition of FAZ assembly causes flagellar detachment, which leads to a rapid and dramatic growth defect, whereas in PCFs the shorter FAZ results in a rearrangement of the cellular architecture, giving rise to epimastigote-like cells with a long unattached flagellum. Differences in cell division between BSFs and PCFs are likely to cause the difference in phenotype between the life cycle stages (Wheeler et al., 2013). In a normal post-mitotic PCF cell the arrangement of the kinetoplasts and nuclei from posterior to anterior is KNKN, whereas in a BSF cell it is KKNN (Fig. 7). In PCF cells the inter-kinetoplast and inter-nucleus distances are greater than in BSF cells, and there is also differential placement of the cytokinesis furrow. In a PCF cell the reduction in FAZ length causes an initial decrease in the inter-kinetoplast distance on the first division, but the inter-nucleus distance remains the same. Hence, the posterior nucleus ends up closer to the posterior kinetoplast and the cell adopts an epimastigote-like morphology (Fig. 7).

In a post-mitotic BSF cell the inter-kinetoplast and inter-nucleus distances are shorter and, therefore, the effect of a shorter FAZ is different. In a dividing BSF cell with a shorter FAZ the inter-kinetoplast distance would be smaller with the inter-nucleus distance remaining the same (Fig. 7). However, as the dividing nucleus does not separate to the same degree as in a PCF cell, the posterior nucleus and kinetoplast would not be sufficiently close to be partitioned together into the daughter cell, especially as the cytokinesis furrow in a dividing BSF cell is positioned closer to the existing posterior pole. Hence, the first division post-induction potentially gives rise to a variety of cell types, depending on the cell-cycle stage of the cells when the FLAM3 RNAi was induced. Three possible scenarios can be envisaged: (i) if a cell had assembled or nearly assembled a full length FAZ it would divide normally; (ii) if a cell had only partially assembled a FAZ, its division might result in a zoid and 1K2N cell; and (iii) if a cell had not begun or had just begun FAZ assembly, it would probably be unable to divide. Another, possibly contributing, factor to the rapid cessation in BSF growth after FLAM3 RNAi might be related to the endomembrane organisation. In PCFs shortening of the FAZ results in movement of the Golgi complex and rearrangement of the endomembrane system; these changes are tolerated, whereas the equivalent changes in BSFs may not be tolerated because a high rate of endocytic and/or exocytic traffic is required (García-Salcedo et al., 2004).

We propose that these differences in mutant phenotypes are a reflection of the deeper evolutionary biology underlying the complex trypanosome life cycle. In this context, the ability of a PCF cell to undergo differentiation to an epimastigote-like configuration suggests that this cell type is structurally engineered to reach the next step in the trypanosome life cycle. In contrast, the BSF cell differentiates to its next phase in its life cycle – which is the trypomastigote form. Hence the BSF cell might not have the intrinsic structural plasticity to undergo a trypomastigote-to-epimastigote transition.

Inhibition of flagellum elongation through ablation of IFT proteins following RNAi produces cells with a much-reduced FAZ length and, hence, flagellum assembly is crucial for FAZ assembly (Kohl et al., 2003). However, we have clearly shown that modulation of FLAM3 levels leads to shortening of the FAZ, independently of flagellum elongation. Therefore, either the extent or the rate of FAZ elongation is likely to provide a route through which transformation between different cellular forms can occur during the life cycle of *T. brucei*. Modulation of FAZ length, therefore, has a crucial role in determining the morphological fate of a cell. Furthermore, by demonstrating that ClpGM6 and FLAM3 are functionally connected, we are taking the first steps towards the integration of proteins that have defined roles within the FAZ.

## MATERIALS AND METHODS

All reagents were purchased from Sigma-Aldrich (Gillingham, UK) unless stated.

### Cells and plasmids

*Trypanosoma brucei* PCF SMOXP9 cells were grown at 28°C in SDM-79 (Life Technologies, Paisley, UK**)** supplemented with 10% FCS and 1 µg/ml puromycin (Poon et al., 2012). BSF SMOXB4 cells were grown at 37°C in HMI-9 (Life Technologies**)** supplemented with 15% FCS and 0.2 µg/ml puromycin (Poon et al., 2012). Cells in logarithmic growth were used for all experiments. BSF 427, single-marker BSF and PCF 29:13 cells were cultured as described elsewhere (Changmai et al., 2013).

A 938-bp region (nucleotides 1004–1941) of the FLAM3 gene was amplified and ligated into p2T7-177 plasmid to create the FLAM3 RNAi-A plasmid (Wickstead et al., 2002). For the FLAM3 RNAi-B plasmid, a 489-bp fragment of the ORF (nucleotides 5069–5557) was amplified and cloned into p2T7-177 (Wickstead et al., 2002). RNAi plasmids were linearised by *Not*I (NEB, MA).

For tagging an endogenous allele of FLAM3 at the N-terminus with eYFP, a region of the FLAM3 5′-UTR and 5′-ORF were inserted into the plasmid pEnT6B-Y (Kelly et al., 2007). *Xho*I (NEB) was used to linearise this plasmid. For N-terminal endogenous tagging with PTP, a fragment (nucleotides 2-503) of the FLAM3 ORF was amplified and cloned into p2678 (Kelly et al., 2007). This resulted in plasmid pCR12 which was linearised using *Spe*I (NEB). For C-terminal endogenous tagging with PTP, a fragment of the FLAM3 ORF lacking the Stop codon (nucleotides 11656-12452) was amplified and cloned into a derivative of pC-PTP-Neo (antibiotic resistance changed to puromycin) (Schimanski et al., 2005). The resulting plasmid pCR13 was linearised using *Xba*I (NEB).

Linearised plasmids were electroporated using the standard procedure (McCulloch et al., 2004). SMOXP9 and 29:13 cells with either FLAM3 RNAi-A or -B plasmids integrated were selected with phleomycin (5 µg/ml). ClpGM6 RNAi cells were produced as described previously using the opposing promoter plasmid (Hayes et al., 2014). FLAM3 and ClpGM6 RNAi were induced with doxycycline (1 µg/ml). SMOXP9 cells transfected with the FLAM3::eYFP tagging plasmid were treated with blasticidin (10 µg/ml); 29:13 cells transfected with pCR12 and pCR13 (PTP::FLAM3 and FLAM3::PTP, respectively) were treated with puromycin (0.5 µg/ml).

BSF 427, SMOXB4 and Singler Marker BSF cells were electroporated using the Amaxa Nucleofector II (Lonza, Slough, UK), program X-001. SMOXB4 cells with the FLAM3::eYFP tagging plasmid stably integrated were selected for with blasticidin (5 µg/ml). BSF 427 cells were transfected with pCR12 and pCR13, and single-marker BSF cells cells with the FLAM3 RNAi-B plasmid and pCR12. Selection was with 0.2 µg/ml phleomycin (FLAM3 RNAi-B) and 0.2 µg/ml puromycin (pCR12 and pCR13).

### Bioinformatics

The FLAM3 protein sequence was interrogated using a variety of online prediction programs. RADAR (http://www.ebi.ac.uk/Tools/pfa/radar/) was used to detect repeats. COILS (http://embnet.vital-it.ch/software/COILS_form.html) was used to detect the presence of coiled coils. The FLAM3 protein sequence was used to search the PFAM domain database for both PFAM-A and PFAM-B domains.

### Immunofluorescence

SMOXP9 cells were washed with PBS and settled onto slides. Cells were then either fixed in 4% (w/v) paraformaldehyde in PBS for 5 min or, to produce cytoskeletons, incubated in PEME with 1% (v/v) Igepal CA-630 for 1 min. Subsequently, the cytoskeletons were fixed in 4% (w/v) paraformaldehyde in PEME for 5 min. After fixation the paraformaldehyde was quenched with 1% (w/v) glycine in PBS for 5 min. Cells were then treated with 0.1% (v/v) Igepal CA-630 for 5 min to permeabilise them. FAZ1, ClpGM6, PFR, flagella connector and BARP were detected using L3B2, anti-ClpGM6, L8C4, AB1 and anti-BARP, respectively (Briggs et al., 2004; Hayes et al., 2014; Kohl et al., 1999; Urwyler et al., 2007). The slides were incubated with DAPI (1 µg/ml) in PBS for 5 min and then washed in PBS. Samples were mounted with either DABCO or Vectashield. SMOXB4 cells expressing eYFP::FLAM3 were washed twice with PBS and resuspended in PBS with Hoechst 33342 (10 µg/ml) and formaldehyde (0.007%). SMOXP9 and SMOXB4 cells were imaged using a Leica DM5500B microscope with a Hamamatsu Orca-ER camera and the images processed and analysed using ImageJ (Schneider et al., 2012) or the cells imaged using an Applied Precision DeltaVision deconvolution microscope system and processed using SoftWoRx software.

BSF 427, single-marker BSF cells and PCF 29:13 cells were fixed for 1 h in either 4% (w/v) formaldehyde at room temperature or in methanol at −20°C. Following permeabilisation (formaldehyde fixation only) in PBS with 0.1% (v/v) Triton X-100, protein-A-tagged FLAM3 was detected by using antibody against protein A (1:5000). The slides were incubated with DAPI (1 µg/ml) in PBS for 5 min and then washed in PBS. The cells were examined under an Axioscope II fluorescent microscope.

### Analysis of FLAM3 signal intensity

A single plane of focus for each cytoskeleton for FLAM3 signal intensity analysis was acquired at room temperature by using a Leica DM5500B (widefield) microscope controlled by the Micromanager software, with 100× objective and Andor Neo 5.5 sCMOS camera with the analysis performed in ImageJ. A 20-pixel-wide line was drawn along the FLAM3 signal in the flagellum and then straightened. The signal intensity for each of the pixels in the region defined by the line was added up and then divided by the length in pixels of the line to give an average measure per unit length of FLAM3 signal in that region. For the uninduced cells (*n*=30) three regions of FLAM3 signal were measured; (1) the total length, (2) the length of the attached flagellum and, (3) the length of the free flagellum. On induced cells (*n*=30), three regions of FLAM3 signal were measured; (1) the total length, (2) a line 50 pixels long that started 30 pixels from the proximal end of the FLAM3 signal and, (3) a line 50 pixels long that started 80 pixels from the distal end of the FLAM3 signal. These values were then normalised relative to the value for the total length FLAM3 signal in the uninduced cells (±s.d.).

### Flow cytometry

Live cells were analysed using the BD Accuri C6 flow cytometer with the 533/30 nm filter. 50,000 events were collected for each sample. Analysis was performed by putting a gate on the forward scatter/side scatter plot where >98% of the parental cells are positioned. The median fluorescent intensity for the resulting events was calculated and then the median fluorescence intensity from the parental cell lines not expressing eYFP::FLAM3 was subtracted from this to give the fluorescence intensity above background. The average fluorescence intensity (±s.d.) above background from three separate experiments was plotted.

### Western blotting

4×10^6^ cell equivalent of whole-cell lysates were loaded into each lane and separated by SDS-PAGE. Proteins were transferred to nitrocellulose by semi-dry transfer. Membranes were blocked overnight at 4°C with blocking buffer (3% (w/v) milk powder in TBS-T). Primary antibody (anti-ClpGM6 1:2000, anti-GFP 1:1000 (Life Technologies, Paisley, UK**)**, anti-FAZ1 (L3B2) neat, anti-PFR (L8C4) 1:1000, and anti-BARP 1:2500) were diluted to the appropriate concentrations and added to the membrane for 1 h at room temperature (Hayes et al., 2014; Kohl et al., 1999; Urwyler et al., 2007). The membranes were washed with blocking buffer and then incubated with the secondary antibody (anti-rabbit HRP 1:5000) in blocking buffer for 1 h at room temperature. The membranes were washed in blocking buffer and antibodies detected by enhanced chemiluminescence.

### Dot blots

5×10^6^ cell equivalent of whole-cell lysates per spot were placed onto a nitrocellulose membrane and allowed to air-dry. The membrane was blocked in 5% milk in PBS for 2 h at room temperature, prior to incubation with the primary antibody overnight at 4°C (anti-Protein A, 1:20,000) or 1 h at room temperature (anti-enolase, 1:10,000; kind gift from Paul Michels). Following two washes in PBS, the membrane was incubated with anti-rabbit HRP secondary antibody (1:2000) for 1 h at room temperature. Proteins were detected by enhanced chemiluminescence.

### Electron microscopy

#### TEM

Cells were fixed in medium supplemented with glutaraldehyde (final concentration of 2.5%) for 3 min, washed with buffered fixative (100 mM sodium phosphate buffer pH 7, 2.5% glutaraldehyde, 3% formaldehyde). The cells were incubated in buffered fixative for a minimum of 2 h at 4°C, washed thoroughly with 100 mM sodium phosphate buffer pH 7 and then post-fixed with 1% osmium tetroxide in 100 mM sodium phosphate buffer for 2 h at 4°C. Cells were then washed with water and stained with 2% aqueous uranyl acetate for 2 h at 4°C in the dark. They were then dehydrated, followed by epoxy resin infiltration. The resin was polymerised overnight at 60°C; then sections were cut and later stained with lead citrate (Reynolds, 1963) for 30 s at room temperature and imaged.

#### SEM

SMOXP9 cells were fixed in medium of a final concentration of 2.5% glutaraldehyde for 2 h at room temperature while shaking. The fixed cells were centrifuged, washed three times with PBS and resuspended in PBS. The cell suspension was allowed to settle onto coverslips for 1 h and adhered cells were then dehydrated. The samples were critical-point dried, sputter coated with gold and imaged.

PCF 29:13 and single-marker BSF cells were fixed in 2.5% glutaraldehyde in 100 mM PBS overnight at 4°C and then spotted onto poly-l-lysine-coated glass cover slips. The cells were post-fixed in 2% osmium tetroxide in 100 mM PBS for 1 h at room temperature and finally washed in the same buffer. After dehydration cells were critical-point dried, coated with gold palladium and imaged.

## Supplementary Material

Supplementary Material
