# Supplementary material for: Modulation of flagellum attachment zone protein FLAM3 and regulation of the cell shape in Trypanosoma brucei life cycle transitions
Source: J Cell Sci. 2015 Aug 15;128(16):3117–30. doi: 10.1242/jcs.171645 (PMC4541047; doi:10.1242/jcs.171645)
Supplement: Supplementary Material [file supp_128_16_3117__index.html]

Supplementary Material 

# Flagellum attachment zone protein modulation and regulation of cell shape in *Trypanosoma brucei* life cycle transitions

## JCS171645 Supplementary Material

- Supplementary Material
