# Supplementary material for: Modulation of flagellum attachment zone protein FLAM3 and regulation of the cell shape in Trypanosoma brucei life cycle transitions
Source: J Cell Sci. 2015 Aug 15;128(16):3117–30. doi: 10.1242/jcs.171645 (PMC4541047; doi:10.1242/jcs.171645)
Supplement: Supplementary Material [file supp_jcs.171645_JCS171645supp.pdf]

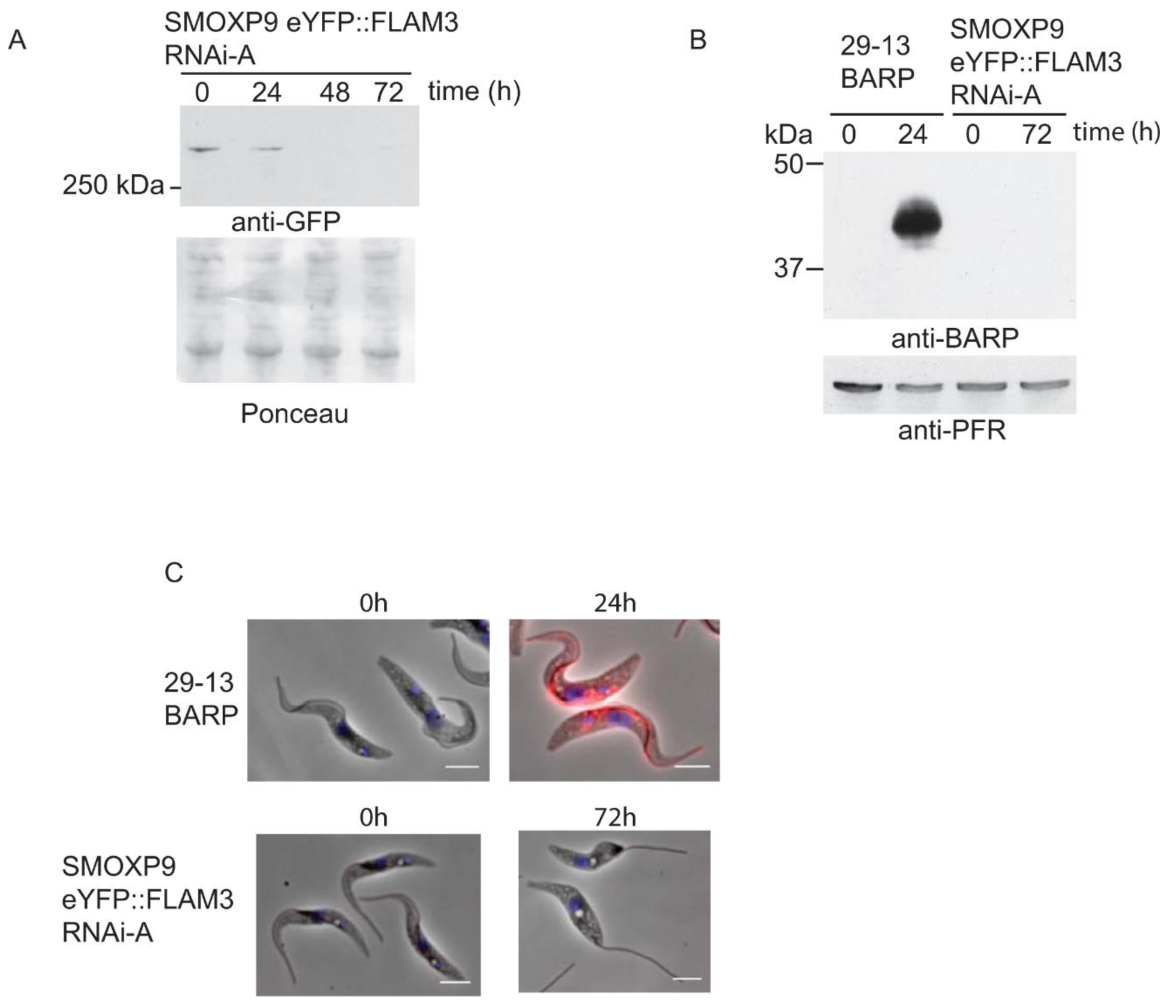

Supplementary Figure 1. RNAi knockdown of FLAM3 causes a reduction in eYFP::FLAM3 expression without expression of the epimastigote marker, BARP.

A) estern blot using the anti-GFP antibody of SMOXP9 eYFP::FLAM3 FLAM3 RNAi-A cells during a FLAM3 RNAi induction time course. Ponceau staining is a loading control. 4 x 106 cell equivalents were loaded per lane.

B) estern blot using the anti-BARP antibody of a 29-13 cell line carrying a doxycycline inducible BARP expression plasmid before and after 24 hours of induction and of the SMOXP9 eYFP::FLAM3 RNAi-A before and after 72 hours of induction. The anti-PFR antibody was used as a loading control. 5 mM bathophenanthroline disulfonic acid, a metalloprotease inhibitor, was added to cultures to be analysed for BARP expression 20 hours before harvesting.

C) Immunofluorescence of 29-13 cell line carrying a doxycycline inducible BA expression plasmid before and after 24 hours of induction and of the SMOXP9 eYFP::FLAM3 RNAi-A before and after 72 hours of induction.5 mM bathophenanthroline disulfonic acid, a metalloprotease inhibitor, was added to cultures to be analysed for BARP expression 20 hours before harvesting. Scale bar, 5 μm.

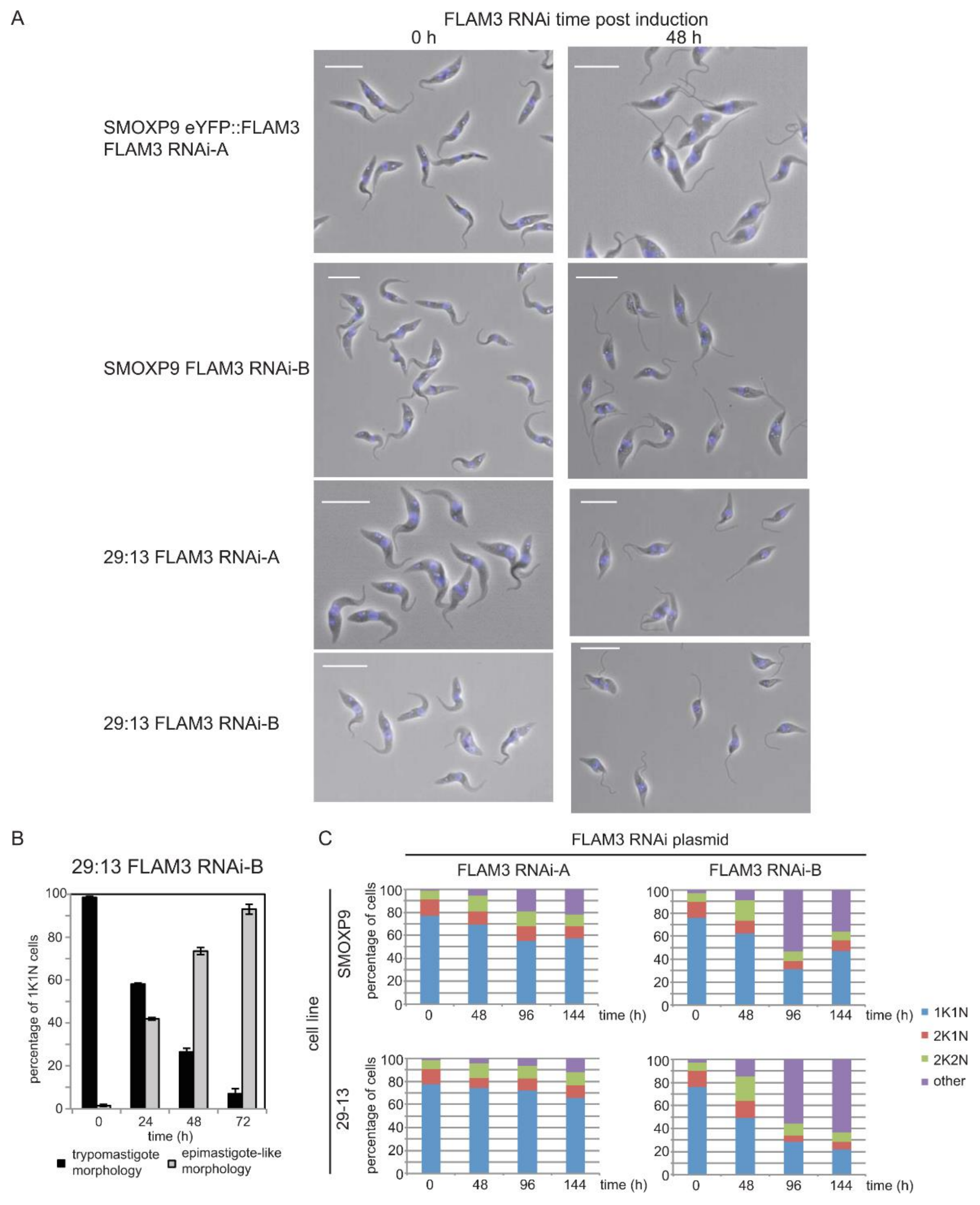

Supplementary Figure 2. Knockdown of FLAM3 causes epimastigote-like appearance.
A) Field of views of the d ferent FLAM3 RNAi cell lines before and after RNAi induction. Phase images of cells overlaid with DAPI (blue) staining the nucleus and kinetoplast. After 48 hours of induction all RNAi cell lines show the same morphology with a long unattached flagellum and the kinteoplast juxtaposed or anterior to the nucleus. Scale bar denotes 10 µm.
B) Grpah showing the percentage of 1K1N cells with a trypomastigote or epimastigote-like morphology during FLAM3 RN induction. 29-13 FLAM3 RNAi-B cells were induced with tetracycline and the morphology of 200 1K1N cells per time point in three independent experiments was assessed. The average percentage was plotted ±SD.
C) Counts of kinteoplast and nucleus for the d ferent FLAM3 RNAi plasmids in the different parental cell lines during a FLAM3 RNAi timecourse. Other category includes zoids (1K0N) and monsters (XKXN).

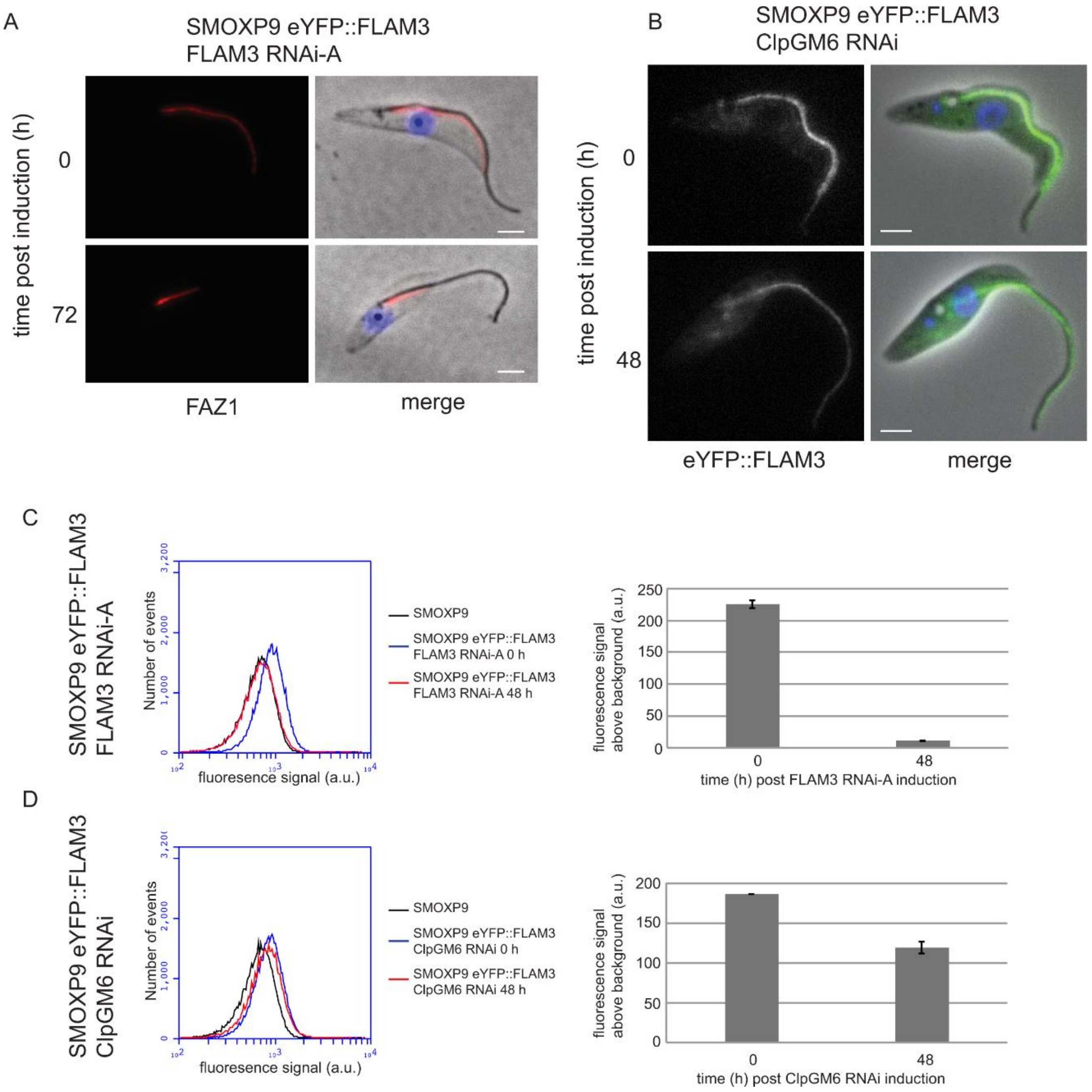

Supplementary Figure 3. A) Knockdown of FLAM3 does not alter FAZ1 expression. Images of cytoskeletons stained for FAZ1 (red) with L3B2 and DNA (blue) with DAPI before and after doxycycline induction. Scale bar denotes 2 μm.

B) Knockdown of ClpGM6 results in a reducton and redistribution of FLAM3. Images of live whole cells wi native eYFP::FLAM3 fluorscent (green) and DNA (blue) stained with Hoescht before and after doxycycline induction. Scale bar denotes 2 μm.

C) Knockdown of eYFP::FLAM3 signal measured by flow cytometry after FLAM3 RNAi A typical example of the flow cytometry data using live SMOXP9 eYFP::FLAM3 FLAM3 RNAi-A cells before and after doxcycline induction for 48 hours. The median fluorescent signal from three inductions was quantified and plotted (±SD) showing a ~90% reduction in eYFP::FLAM3 signal.

D) Knockdown of eYFP::FLAM3 signal measured by flow cytometry after ClpGM6 RNAi A typical example of the flow cytometry data using live SMOXP9 eYFP::FLAM3 ClpGM6 RNAi cells before and after doxcycline induction for 48 hours. The median fluorescent signal from three inductions was quantified and plotted (±SD) showing a ~30% reduction in eYFP::FLAM3 signal.

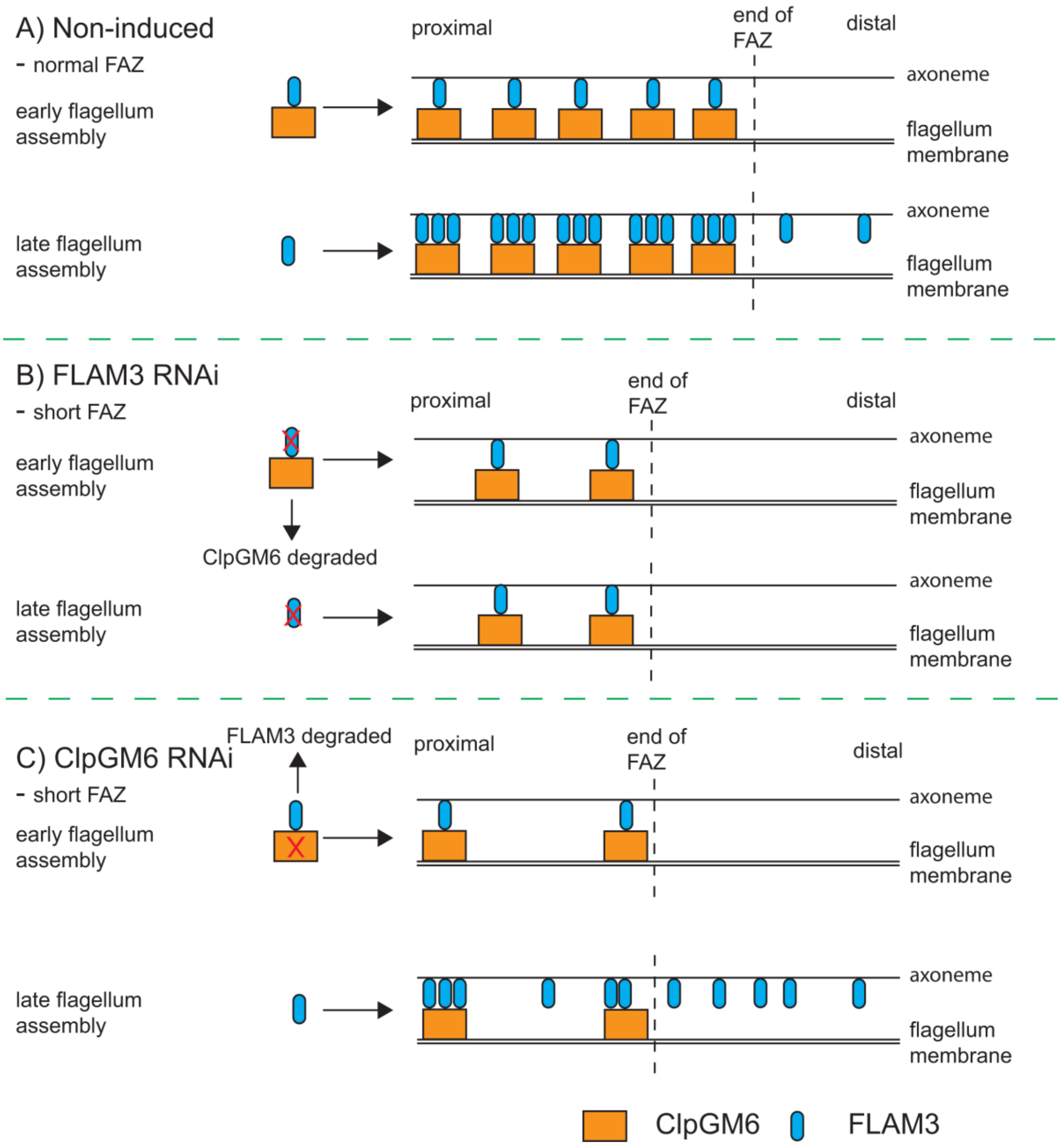

Supplementary Figure 4. Model for FLAM3 and ClpGM6 assembly into the FAZ with and without FLAM3 or ClpGM6 RNAi.
A) An initial complex of FLAM3 and ClpGM6 is formed, which is assembled into the growing FAZ. A later addition of FLAM3 then occurs with the protein integrating into the existing ClpGM6 FLAM3 complexes and any FLAM3 excess is distributed along the flagellum.
B) On FLAM3 RNAi the complex is unable to form and is degraded, leading to reduced levels of ClpG expression.
C) On ClpGM6 RNAi the complex is unable to form and is degraded, leading to a reduction in FLAM3 The loss of ClpGM6 does not effect the later addition of FLAM3 to the flagellum.
